# A Dynamic DEA Analysis of Health Output Efficiencies of Cities and Counties in Taiwan

**DOI:** 10.3390/ijerph20064674

**Published:** 2023-03-07

**Authors:** Jin-Li Hu, Min-Yueh Chuang, Shang-Ho Yeh

**Affiliations:** Institute of Business and Management, College of Management, National Yang Ming Chiao Tung University, Taipei City 10044, Taiwan

**Keywords:** health output efficiency, undesirable model, dynamic DEA, disaggregated efficiency

## Abstract

This research utilizes the dynamic slack-based measure (DSBM) model to evaluate health output efficiencies in Taiwan’s administrative districts from 2014 to 2018. To measure health output efficiency, it adopts four input variables, one output variable, and one carry-over (exercise expenditure). This paper includes both public goods in totals and private goods per capita as the inputs of health output. Empirical results indicate that health output efficiencies in the south and east areas relatively lower. Lastly, the overall efficiency of urban areas may not be better than that in non-urban areas.

## 1. Introduction

The impacts of the COVID-19 pandemic on life, health, society, and the economy have demonstrated the importance of healthcare system efficiency and the level of preparation needed to reduce its negative effects while preserving public health [1,2]. A healthy population is vital to every country and region because it strongly impacts social and economic progress [2,3].

The literature has estimated factors affecting public health from a variety of indicators [4], such as life expectancy at birth and mortality rates [5], socioeconomic, lifestyle, and environmental factors [6], exercising regularly [7], healthcare expenditure [8,9,10], and public health expenditure [11]. Therefore, decision-makers of health policies are becoming increasingly concerned with the overall performance of the healthcare system and the improvement factors that influence public health [8,11,12]. Their ultimate purpose for the healthcare system is to maximize the population’s health and alleviate health inequalities [3]. Thus, the public and private health sectors are often viewed as being critical roles of resources to improve the efficiency of healthcare resource utilities [13].

The condition of any healthcare system is an important issue, and decision-makers in many countries have formulated policies that aim at improving the system and eliminating health inequalities [1,3,14,15]. Under ideal circumstances, everyone should be able to benefit from the advantageous conditions in an environment of good health [16]. Accordingly, one of the most important steps in the study of a healthcare system is to find the relative factors and provide useful information on which to base policy decisions regarding life expectancy, the development of healthcare-related industries, and where to invest medical expenditures [5]. The efficiency model assists in such evaluations. In addition to emitting desirable outputs, the production process may also generate undesirable products or bad output [17]. However, few studies related to health efficiency have investigated the issue of bad outputs and their negative impact [18]. Distinguishing between good and bad outputs related to healthcare efficiency should certainly not be neglected.

From the viewpoint of the resource-based theory (RBT), Barney [19] argued that a firm can achieve a sustainable competitive advantage by exploiting its strategic resources. According to RBT, a company can gain a long-term and sustainable competitive advantage through the accumulation and cultivation of its internal resources and capabilities [19]. In relation to health, Ngandu et al. [20] found that exercise has positive effects in terms of preventing or relieving mental illness in the long term. It is also essential for preventing lifestyle-related diseases and improving health [21]. Exercise is known not only to develop physical fitness, but also to alleviate the incidence of disease [20,22] and improve health [21]. Thus, looking at health output efficiency from the viewpoint of RBT, exercise is one kind of resource affecting a population’s health in the long run. It is a type of health capital [4] that enhances the health output of people and should be a long-term consideration for evaluating the health outcome.

Most policymakers need quantitative information to evaluate effective and targeted policies [23]. While data envelopment analysis (DEA) has been widely adopted to evaluate the efficiency of healthy industries [24,25], there have been few long-term studies dealing with measuring health output efficiency, where carry-over activities between two consecutive terms are a concern. Carry-over plays a critical role in estimating the efficiency of decision-making units (DMUs), not only in each period, but also over the whole term [17]. There are some methods in DEA that consider long-time issues, such as the Malmquist index [17,26] and window analysis [17], but they do not consider carry-over activities very much. The interdependencies between consecutive periods are also not considered in traditional DEA models [27], which can be problematic when considering long-term health efficiency. For example, it is found that the level of exercise in the immediately preceding period modifies the efficiency assessment in subsequent periods [27,28].

To improve the measurement of health output efficiency, this research therefore introduces a carry-over variable, denoted as the exercise variable. This has not been considered in previous studies to estimate health output efficiency. Finally, this study adopts the DSBM model and incorporates a carry-over activity to analyze long periods [17].

The Taiwan government’s expenditure in the healthcare field has been increasing in recent years, and thus, healthcare efficiency and its influencing factors are noteworthy problems [7,24,25]. Understanding what determines health output efficiency is significant from the viewpoint of health policy [7,10]. Hence, this study aims to strengthen our understanding of the determinants of health output efficiency. First, in contrast to earlier studies, it uses both total efficiency and disaggregated efficiency at the county and city levels in Taiwan to explain health output efficiency at the local level, because county- and city-level data are still macroeconomic, but measured at a much finer scale than at the country level. Second, in contrast to previous studies, we adopt a long-term method that allows for measuring period-specific efficiency based on a longer period of time.

The rest of this paper is as follows. Section 2 provides a literature review of health status determinants. Section 3 presents our health production function model. Section 4 describes the data and empirical specification. Finally, Section 5 discusses the empirical results and outlines the main conclusions and policy implications.

## 2. Literature Review

Grossman [4] developed a health production function model, in which healthcare is regarded as a durable capital good that can be used to produce a healthy life. Thus, every independent individual has a fixed health stock that will gradually decrease with age. Progress can be made with health performance and improving health by increasing health investment behavior. However, the conditions that cause differences in health efficiency are very diverse, including medical innovation, public health expenditure [11], health expenditure, environment, economic development [24], urban green spaces [29], health and medical expenditure, salary, level of education [5], and physical activity [30]. Some studies also noted the effects of regular physical activity on health to be long-lasting [20,21].

### 2.1. The Relationship between the Healthcare System and Health

Berger and Messer [8] analyzed data from 20 OECD countries for the years 1960–1992. Their findings indicated that mortality rates depend on healthcare expenditure and health insurance coverage. Mackenbach et al. [9] explored what accelerated the rise in life expectancy in the Netherlands in 2002. They found that healthcare expenditure rose quickly after 2001, and the healthcare system was the reason for the decline in mortality. In another study, Keng and Sheu [15] analyzed the effect of increased public healthcare expenditure with the advent of National Health Insurance (NHI) in Taiwan on mortality and health self-assessment. Their results indicated that the introduction of NHI led to a reduction in the mortality rate in unhealthy groups as well as in less privileged groups with vulnerable groups.

Average life expectancy not only represents the health of the people and the growth and decline of life, but also denotes a region’s social and economic well-being [31,32]. Longevity is the output of the health production function, where medical innovation and public health expenditure contribute to greater longevity [11]. Compared to mortality, the chance of people’s survival is greater than the chance of death. Thus, people pay more attention on how to stay healthy and make life better instead of focusing on how to avoid death [33]. Accordingly, this study adopts life expectancy as the health output efficiency variable.

### 2.2. Health Expenditure

The basic objective of a healthcare system is to meet a country’s health needs the most efficiently and to keep it financially sustainable [34]. Normally, health expenditures are divided into public and private health expenditures [35]. The financing of healthcare through public ways is an important input and has implications for the health policy goals of equity, efficiency, and sustainability. Novignon, Olakojo, and Nonvignon [36] used panel data from 1995 to 2010 covering 44 countries in sub-Saharan Africa. Their results showed that healthcare expenditure significantly influences health status. Rad et al. [35] indicated that public health expenditures in eastern Mediterranean countries improved health outcomes. As there is no evidence of public expenditure crowding out private expenditure [37], this study examines both public and private healthcare expenditures by adopting them as input variables.

### 2.3. Healthcare Professionals

In addition to health expenditures, the healthcare system also needs to consider medical healthcare factors, such as healthcare professionals. Medical healthcare systems play an important role in healthcare so that people can maintain and improve their health. Health expenditure is viewed as an input by the healthcare system, while healthcare professionals represent the input factor of human capital [38]. However, healthcare systems face a number of challenges, one of which is an uneven distribution across regions of healthcare professionals [39]. Therefore, this study also takes into account healthcare professionals as an input to evaluate regional health output efficiencies.

### 2.4. Income

Some studies have presented the relationship between income and health [40]. Jones and Wildman [41] investigated the relationship between income and health, demonstrating that income affects health. Barlow and Vissandjee [42] reported that income level, education, fertility, and location are important for life expectancy. The reproduction of the income gradient in health-related quality of life through social networking time mainly persists in mental health aspects [43]. Therefore, income is assumed to be related to the quality of life [43], health [41], and life expectancy [42].

### 2.5. Exercise Expenditure

Another factor that has an important impact on life expectancy is exercise. King et al. [44] considered exercise in their analysis of life expectancy and found that both community-based and home-based exercise training programs improve fitness in older adults. Physical activity has positive effects [21]. People who maintain exercise habits are less likely to use medical services, and they spend less in inpatient expenses [7]. Exercise is therefore considered one of the factors impacting healthy output efficiency in the long term, but it takes a long time to see the overall effect [20,22,30]. Any exploration of health output efficiency needs to consider factors that have long-term effects on variations in health status. However, these factors have been considered only in longitudinal studies, while cross-sectional or carry-over issues in the long term, such as exercise, have not been targeted.

With the gradual increase in healthcare expenditures in various countries around the world, many medical policy decision-makers and economists are concerned about the performance of healthcare systems [45]. How to contain escalating healthcare spending has become a big challenge in many countries [7]. The implementation of health insurance for everyone in Taiwan has received support for its fairness of access to medical care, meaning that everyone has the same opportunities for medical care, whether they are rich or poor [46]. Therefore, this study utilizes Taiwan as the sample for further analysis of the differences in health output efficiency between cities and counties. The input items include public sector medical expenditure, private sector medical expenditure, number of healthcare professionals, and income. With respect to output, this study employs life expectancy, and the carry-over variable is exercise expenditure.

## 3. Methodology

DEA measures efficiency with multiple input and output variables. Tone and Tsutsui [17] applied carry-over variables in a dynamic DEA model designed to make estimates over several time periods. There are n decision-making units (DMUs) over T terms. In each term t, each DMU has its own inputs and outputs along with the carry-over to the next term *t* + 1. They classified carry-over activities, called links, into four categories: desirable ($z^{good}$), undesirable ($z^{bad}$), discretionary ($z^{free}$), and non-discretionary ($z^{fix}$).

This study observes n DMUs (counties and cities) over T terms. In each term t, each DMU uses its respective four inputs (public sector medical expenditure, private sector medical expenditure, healthcare professionals, and income) to produce one desirable output (life expectancy). The link variables connect consecutive terms (1, …, *t* − 1, *t*, *t* + 1, *T*). Herein, the level of exercise expenditure available for each DMU in term t determines the exercise expenditure in the immediately succeeding term, *t* + 1, and is determined by the exercise expenditure in the immediately preceding term, *t* − 1.

The production possibilities denoted by $\left\{x_{it}\right\}$, $\left\{y_{it}\right\}$, $\left\{z_{it}^{good}\right\}$, and $\left\{z_{it}^{bad}\right\}$ are given by:

$$x_{it}\geq \sum_{j=1}^{n}x_{ijt}\lambda_{j}^{t},\;\;\;\left(i=1,\ldots ,m;t=1,\ldots ,T\right);$$

$$y_{it}\leq \sum_{j=1}^{n}y_{ijt}\lambda_{j}^{t},\;\;\;\left(i=1,\ldots ,s;t=1,\ldots ,T\right);$$

$$z_{it}^{good}\leq \sum_{j=1}^{n}z_{ijt}^{good}\lambda_{j}^{t},\;\;\;\left(i=1,\ldots ,ngood;t=1,\ldots ,T\right);$$

$$z_{it}^{bad}\geq \sum_{j=1}^{n}z_{ijt}^{bad}\lambda_{j}^{t},\;\;\left(i=1,\ldots ,nbad;=1,\ldots ,T\right);$$

$$\lambda_{j}^{t}\geq 0,\;\;\left(j=1,\ldots ,n;t=1,\ldots ,T\right);$$

(1) $$\sum_{j=1}^{n}\lambda_{j}^{t}=1,\;\left(t=1,\ldots ,T\right).$$

The continuity of carry-over links between terms *t* and *t* + 1 is guaranteed by the following condition, where α is the standard symbol for good and bad links:

(2) $$\sum_{j=1}^{n}z_{ijt}^{\alpha }\lambda_{j}^{t}=\sum_{j=1}^{n}z_{ijt}^{\alpha }\lambda_{j}^{t+1},\;\left(\forall i;t=1,\ldots ,T-1\right).$$

Note that the summation of peer weights (λ) in a term (*t*) is unity, indicating that this is a variable-returns-to-scale (VRS) model. This restriction is important for the dynamic model since it connects the activities of terms t and *t* + 1.

Using these equations for production, we express $DMU_{o}\left(o=1,\ldots ,n\right)$ as follows:

$$x_{iot}=\sum_{j=1}^{n}x_{ijt}\lambda_{j}^{t}+s_{it}^{-},\;\left(i=1,\ldots ,m;t=1,\ldots ,T\right);$$

$$y_{iot}\leq \sum_{j=1}^{n}y_{ijt}\lambda_{j}^{t}-s_{it}^{+},\;\left(i=1,\ldots ,s;t=1,\ldots ,T\right);$$

$$z_{iot}^{good}=\sum_{j=1}^{n}z_{ijt}^{good}\lambda_{j}^{t}-s_{it}^{good},\;\left(i=1,\ldots ,ngood;t=1,\ldots ,T\right);$$

$$\sum_{j=1}^{n}\lambda_{j}^{t}=1,\;\left(t=1,\ldots ,T\right);$$

(3) $$\lambda_{j}^{t}\geq 0,\;s_{it}^{-}\geq 0,\;s_{it}^{+}\geq 0,\;s_{it}^{good}\geq 0.$$

Here, $x_{iot}$ indicates inputs, which are public sector medical expenditure, private sector medical expenditure, number of healthcare professionals, and income; $y_{iot}$ denotes the desirable output, which is life expectancy; $z_{iot}^{good}$ is a good carry-over, which is exercise expenditure;$s_{it}^{-}$ denotes input slack; $s_{it}^{+}$ denotes desirable output slack; and $s_{it}^{good}$ denotes desirable output slack.

The output-oriented overall efficiency $\tau_{o}^{*}$ with the good link (exercise expenditure) is represented by:

(4) $$\frac{1}{\tau_{o}^{*}}=\max\frac{1}{T}\sum_{t=1}^{T}[1+\frac{1}{l+ngood}\left(\sum_{i=1}^{l}s_{it}^{+}+\sum_{i=1}^{ngood}\frac{s_{it}^{good}}{z_{iot}^{good}}\right)].$$

Using the optimal solution $\left\{\lambda^{t*}\right\}$, $\left\{s_{t}^{-*}\right\}$, $\left\{s_{t}^{+*}\right\}$, and $\left\{s_{t}^{good*}\right\}$, the output-oriented term efficiency can be defined as follows:

(5) $$\tau_{ot}^{*}=\frac{1}{1+\frac{1}{l+ngood}\left(\sum_{i=1}^{l}\;\frac{s_{iot}^{+*}\;}{y_{iot}}+\sum_{i=1}^{ngood\;\;\;\;}\frac{s_{iot}^{good*}}{z_{iot}^{good}}\right)},\;\left(t=1,\ldots ,T\right).$$

The output-oriented efficiency during period $\left(\tau_{ot}^{*}\right)$ is a harmonious mean of the efficiencies of periods $\left(\tau_{ot}^{}\right)$, as demonstrated below:

(6) $$\frac{1}{\tau_{o}^{*}}=\frac{1}{T}\sum_{t=1}^{T}\frac{1}{\tau_{ot}^{*}}$$

The overall efficiency during the period $\left(\theta_{o}^{*}\right)$ is the weighted average of the term efficiencies $\theta_{ot}^{*}$ as demonstrated below:

(7) $$\theta_{o}^{*}=\frac{1}{T}\sum_{t=1}^{T}\theta_{ot}^{*}.$$

This study uses the DEA model to obtain a scalar measure of relative efficiency for 22 cities and counties from different geographic regions in Taiwan. All statistical data come from the public government’s statistics and annual reports. However, data for two counties are excluded, because of missing values. Hence, the final sample includes data for 20 cities and counties for the years from 2014 to 2018, including Keelung City, Taipei City, New Taipei City, Taoyuan City, Hsinchu County, Hsinchu City, Miaoli County, Taichung City, Changhua County, Yunlin County, Chiayi County, Chiayi City, Nantou County, Tainan City, Kaohsiung City, Pingtung County, Yilan County, Hualien County, Taitung County, and Penghu County.

The data enter a dynamic model with five periods, utilizing four inputs, one output, and one carry-over. The inputs are public sector medical expenditure, private sector medical expenditure per capita, healthcare professionals, and income per capita. The final output item is life expectancy. The public sector medical expenditure and health professionals data are in regional totals because they are public goods commonly consumed by the residents in an administrative region, whereas the private sector medical expenditure data are per capita because they are treated as private goods consumed individually. The carry-over variable is exercise expenditure (desirable), which is in regional totals because of its public good property. All monetary variables have been deflated into real variables in the 2014 base by the GDP deflators of Taiwan. Some studies suggest the number of DMUs should be at least twice the number of input and output [47,48]. Therefore, this proportion is deemed acceptable in this study. The variable definitions are shown in Table 1.

## 4. Empirical Analysis

The scope of this study spans from 2014 to 2018, or a total of five years. All data are based on the year 2014. The GDP deflator is used to convert data into real variables. When using DEA, it is necessary to consider the isotonicity of both inputs and outputs [49]. Note that there is a positive correlation between input items and output items. Table 2 lists the results of the correlation analysis conducted by using the average number of input and output items for the five years from 2014 to 2018. We see a positive correlation between inputs and intended output, which runs in line with the isotonicity conditions [33,49]. The correlation coefficients between inputs and the output are shown in Table 2.

Table 3 presents the descriptive statistics. It shows that the average public sector medical expenditure per year is 1,042,868.960, with a maximum of 5,016,109.934 in Taipei City and a minimum of 161,835.873 in Chiayi City. The average private sector medical expenditure is 38,967.154, with a maximum of 45,630.157 in Yilan County and a minimum of 31,127.354 in Taoyuan City. The average number of healthcare professionals is 119.737 with a maximum of 229.974 in Chiayi City and a minimum of 73.930 in Penghu County. The average income is 373,981.240 with a maximum of 543,513.955 in Taipei City and a minimum of 303,502.068 in Chiayi County. The average life expectancy is 79.321 with a maximum of 83.418 for Taipei City and a minimum of 75.284 in Taitung County. The average exercise expenditure is 673,221,604.211 with a maximum of 3,961,894,013.620 in Taipei City and a minimum of 67,149,016.798 in Nantou County.

Table 4 shows the overall efficiency scores and ranks obtained for the 20 cities and counties with the dynamic DEA model. The results indicate that 10 DMUs were operating at the relatively high efficiency of 1 for the period from 2014 to 2018, including Keelung City, New Taipei City, Taoyuan City, Hsinchu County, Miaoli County, Changhua County, Yunlin County, Chiayi City, Nantou County, and Penghu County. The efficiency performance of these 10 DMUs is thus better than the other 10 DMUs. The results also indicate that Pingtung County (efficiency score = 0.530), Hualien County (efficiency score = 0.583), and Yilan County (efficiency score = 0.731) have the lowest 3 output efficiencies of the 20 cities/counties.

Overall technical efficiency does not take into account the differences in disaggregated efficiency scores. Hence, this study uses the disaggregated efficiency score to explore the different input and output variables over five years [50]. The output-oriented DEA model and concepts from Hu and Chang [50] are adopted to calculate and identify the optimal output of inefficient DMUs through linear programming so as to estimate the efficiency performance of the output variables. When the actual output level of a DMU is equal to the target output level, then disaggregated efficiency = 1; otherwise, when it shows higher inefficiency, the disaggregated efficiency is closer to 0 [50].

In the formulation, t represents the *t*th year, and i represents the *i*th DMU. The disaggregated efficiency (i,t) of desirable (exercise expenditure) output variables is:

(8) $$0\leq \frac{\;\mathrm{Actual}\;\mathrm{Output}\left(i,\;t\right)}{\mathrm{Targe}\;\mathrm{Output}\left(i,\;t\right)}\leq 1.$$

Table 5 lists the disaggregated efficiencies of the desirable variable (exercise expenditure) for the 20 cities and counties from 2014 to 2018, which can be adopted to analyze and discuss the main causes affecting a region’s inefficiency. Given the overall operating conditions detailed in the previous section, the disaggregated efficiency for all their output variables is 1 in certain years of the study period. The five cities and counties with the lowest disaggregated efficiencies for exercise expenditure from 2014 to 2018 are Pingtung County, Hualien County, Yilan County, Tainan City, and Kaohsiung City. Table 5 summarizes the disaggregated output scores of counties/cites that still have room for improvement.

From the perspective of the term efficiency (see Table 4), it is found that poor efficiency performance is even in urban areas. Therefore, this study further explores whether there is a difference between urban and non-urban areas. Following Taiwan’s Local Institutional Law, this study classifies a population of more than 1.25 million as living in an urban area. The cities or counties with a population between 300,000 and 1.25 million as well as the remaining counties and cities are called non-urban areas. Therefore, for urban areas, we refer to the six municipalities directly under the central government, and non-urban areas cover three cities (Keelung City, Hsinchu City, Chiayi City) along with the remaining eleven counties (see Table 6).

To assess the relevancy of the statistical significance in the difference between urban and non-urban areas from 2014 to 2018, this study uses the non-parametric Mann-Whitney U test. The results appear in Table 7. It shows that even if urban areas have more medical resources, their overall efficiency may not be better than that of non-urban areas.

## 5. Conclusions

This study applies the DSBM model to measure the health output efficiencies in Taiwan administrative districts from 2014 to 2018. The results show the differences in health output efficiencies of various cities and counties in terms of overall efficiency. In particular, administrative districts with poor health output efficiencies are mostly concentrated in the southern and eastern regions. They include both urban (Tainan City and Kaohsiung City) and non-urban (Pingtung County, Yilan County, and Hualien County) areas. This result is similar to Chiu and Hsu [46] and Kreng and Yang [45], since they indicate that some eastern and non-urban areas are less developed than other areas in Taiwan. From the view of disaggregated efficiency, these five are at the bottom of health output efficiencies among the twenty cities and counties. The result of average disaggregated efficiency for the exercise expenditure indicates that Pingtung County, Yilan County, and Hualien County still have the lowest disaggregated efficiency, which implies much room for improvement.

Kreng and Yang [45] indicate that most medical resources are centralized in Taipei City, and this is consistent with our results. Taipei City is an urban area, and its average values of every index are higher than the total averages. The city’s average values for income and exercise expenditure are the highest, but its health output efficiencies are behind those of other counties and cities from the years 2014 to 2018. Penghu County and Nantou County are rural areas, and some of their average index values are lower than the total averages of other counties and cities, but their health output efficiencies for the years 2014 to 2018 are good. Clearly, administrative districts with sufficient medical equipment or a high degree of urbanization do not necessarily show higher health output efficiencies.

The results show the uneven distribution of medical resource efficiency in Taiwan—including Keelung City, New Taipei City, Taoyuan City, Hsinchu County, Miaoli County, Changhua County, Yunlin County, Chiayi City, Nantou County, and Penghu County. Therefore, it is necessary to increase the utilization of medical resources to raise the efficiency of each county and city and also to promote medical utilization so as to avoid medical waste by improving resource relocation and medical policy. Accordingly, some policy suggestions are offered. (i) Although healthcare resources are generally concentrated in wealthier, more populous regions, these areas do not always present higher health output efficiency. Hence, the government should initiate different policies to reallocate health resources. (ii) The maldistribution of healthcare resources also creates a rural–urban gap, as indicated by the uneven distribution of public and private sector medical expenditures and healthcare professionals between rural and urban areas. Accordingly, medical healthcare resources in some regions are lacking. More resources need to be developed in the medical field to upgrade health output efficiency, shrink the gap, and balance the distribution.

Based on the above conclusions, this study suggests policymakers consider features of inefficient regions that might cause differential impacts of health output efficiency between urban and non-urban areas. Therefore, a better understanding of the differences in health output efficiency between urban and non-urban areas is important. Following this viewpoint, health expenditure decisions need to avoid excessive resource outputs.

Although there are advantages to using the dynamic DEA model, the DEA results are heavily dependent on the selection of analytical variables. Accordingly, a different set of dimensions may lead to different analysis results. As more information and data become available, further research can examine the association of other exogenous health factors—for instance, environmental factors, and regional differences. Future studies can also consider including mediating variables and moderating variables to measure health output efficiencies.

## Figures and Tables

**Table 1:** Variable Definitions.

Variable Name		Variable Definition	Unit
Input Variables	Public sector medical expenditure in total	Total government sector expenditures designated for medical care for the current year, as public goods	Thousand NT$ in 2014
	Private sector medical expenditure per capita	Average medical expenditure per household in each county and city/average number of individuals per household, based on 2014 data, as private goods	NT$ in 2014
	Healthcare professionals in total	Number of practicing healthcare professionals, as public goods	People
	Real income per capita	Average regular income per household in each county and city/average number of individuals per household, as private goods	NT$ in 2014
Output Variable	Life expectancy per capita	Average remaining life in each county and city	Years
Carry-over Variable	Exercise expenditure in total	Public sports funds of counties and cities as public goods	NT$ in 2014

Notes: The per capita income and per capita medical expenditures of all counties and cities are based on 2014 data. The GDP deflator is used to convert the actual income per capita and real private sector medical expenditures of the counties and cities.

**Table 2:** Correlation coefficients between inputs and outputs.

Variable	1	2	3	4	5
1. Public sector medical expenditure	1				
2. Private sector medical expenditure	0.118	1			
3. Healthcare professionals	0.359	0.264	1		
4. Per capita real income	0.583 **	0.215	0.433 **	1	
5. Life expectancy	0.535 **	0.002	0.262 **	0.717 **	1

Note: ** is *p* < 0.05.

**Table 3:** Descriptive statistics.

Region	Public Sector Medical Expenditure	Private Sector Medical Expenditure	Healthcare Professionals	Income	Life Expectancy	Exercise Expenditure
Keelung City	303,943.166	40,921.165	108.324	380,053.090	79.678	157,723,379.159
Taipei City	5,016,109.934	43,332.437	195.490	543,513.955	83.418	3,961,894,013.620
New Taipei City	1,843,125.920	33,392.196	85.582	402,599.242	81.054	1,475,044,255.602
Taoyuan City	1,164,432.628	31,127.354	115.518	397,479.089	80.538	1,516,634,870.276
Hsinchu County	369,445.201	34,743.803	75.392	441,312.176	80.018	398,399,035.314
Hsinchu City	250,166.010	45,182.503	136.614	487,158.838	80.806	168,643,218.336
Miaoli County	474,710.762	36,819.719	82.258	343,237.764	79.064	263,071,850.518
Taichung City	1,802,338.756	38,384.257	136.590	398,528.211	80.186	1,317,647,142.145
Changhua County	679,473.918	32,166.639	107.296	306,865.085	79.946	492,215,567.533
Yunlin County	507,238.275	41,272.523	92.082	314,637.687	78.180	324,501,962.933
Chiayi County	500,874.537	42,825.388	100.108	303,502.068	78.486	125,903,367.247
Chiayi City	161,835.873	37,320.679	229.974	382,698.087	79.930	180,092,848.757
Nantou County	438,141.504	39,129.874	91.414	308,143.820	78.264	67,149,016.798
Tainan City	1,166,959.024	37,274.587	130.434	342,762.945	79.642	634,959,270.257
Kaohsiung City	3,176,706.825	42,300.055	139.372	391,237.443	78.992	1,541,021,200.093
Pingtung County	766,196.765	36,261.399	105.64	319,904.567	76.982	239,525,601.993
Yilan County	507,238.275	45,630.157	120.624	367,873.094	79.554	295,075,887.526
Hualien County	386,408.986	43,551.361	159.702	354,677.250	76.618	145,696,183.438
Taitung County	575,836.082	36,278.866	108.386	338,179.322	75.284	71,239,017.974
Penghu County	766,196.765	41,428.111	73.930	355,261.062	79.774	87,994,394.698
Min	161,835.873	31,127.354	73.930	303,502.068	75.284	67,149,016.798
Max	5,016,109.934	45,630.157	229.974	543,513.955	83.418	3,961,894,013.620
Total Avg	1,042,868.960	38,967.154	119.737	373,981.240	79.321	673,221,604.211
STD	11,180,581.182	4267.428	39.588	61,849.821	1.752	9,931,388,701.300

Note: The highest and lowest values are underlined.

**Table 4:** Term efficiency.

Region	2014	2015	2016	2017	2018	Avg	Rank
Keelung City	1	1	1	1	1	1	1
Taipei City	0.853	1	1	1	1	0.971	13
New Taipei City	1	1	1	1	1	1	1
Taoyuan City	1	1	1	1	1	1	1
Hsinchu County	1	1	1	1	1	1	1
Hsinchu City	1	0.998	1	1	1	1	11
Miaoli County	1	1	1	1	1	1	1
Taichung City	0.847	0.898	0.889	0.722	1	0.871	14
Changhua County	1	1	1	1	1	1	1
Yunlin County	1	1	1	1	1	1	1
Chiayi County	1	1	1	0.983	1	0.997	12
Chiayi City	1	1	1	1	1	1	1
Nantou County	1	1	1	1	1	1	1
Tainan City	0.970	1	0.372	0.988	0.598	0.786	17
Kaohsiung City	0.782	0.908	1	1	0.512	0.840	16
Pingtung County	0.242	0.892	0.305	0.493	0.718	0.530	20
Yilan County	0.674	0.967	0.382	0.910	0.724	0.731	18
Hualien County	0.591	1	0.346	0.575	0.406	0.583	19
Taitung County	0.527	1	1	1	1	0.905	15
Penghu County	1	1	1	1	1	1	1
Avg.	0.874	0.983	0.865	0.934	0.8978	0.886	
Max	1	1	1	1	1	1	
Min	0.242	0.892	0.305	0.493	0.4056	0.418	
St Dev	0.211	0.037	0.265	0.151	0.192	0.182	

Note: The values for the three most inefficient regions are underlined.

**Table 5:** Disaggregated efficiency of desirable output (Exercise expenditure).

DMU	2014	2015	2016	2017	2018	Avg	Rank
Keelung City	1	1	1	1	1	1	1
Taipei City	1	1	1	1	1	1	13
New Taipei City	1	1	1	1	1	1	1
Taoyuan City	1	1	1	1	1	1	1
Hsinchu County	1	1	1	1	1	1	1
Hsinchu City	1	1	1	1	1	1	11
Miaoli County	1	1	1	1	1	1	1
Taichung City	0.735	0.866	0.800	0.618	1	0.860	14
Changhua County	1	1	1	1	1	1	1
Yunlin County	1	1	1	1	1	1	1
Chiayi County	1	1	1	1	1	1	12
Chiayi City	1	1	1	1	1	1	1
Nantou County	1	1	1	1	1	1	1
Tainan City	1	1	0.231	0.977	0.426	1	17
Kaohsiung City	0.756	1	1	1	0.344	0.881	16
Pingtung County	0.138	0.805	0.180	0.335	0.560	1.170	20
Yilan County	0.525	0.937	0.242	0.837	0.567	0.806	18
Hualien County	0.421	1	0.209	0.422	0.254	0.879	19
Taitung County	0.377	1	1	1	1	0.910	15
Penghu County	1	1	1	1	1	1	1

**Table 6:** Urban and non-urban cities and counties.

Urban	Non-Urban
Taipei City	Keelung City
New Taipei City	Hsinchu County
Taoyuan City	Hsinchu City
Taichung City	Miaoli County
Tainan City	Changhua County
Kaohsiung City	Yunlin County
	Chiayi County
	Chiayi City
	Nantou County
	Pingtung County
	Yilan County
	Hualien County
	Taitung County
	Penghu County

**Table 7:** Mann-Whitney U test of the urban and non-urban health efficiency scores.

Year	2014	2015	2016	2017	2018
*p*-value	0.547	0.659	0.841	1.000	0.659

## Data Availability

All data presented in this article can be made available from the corresponding author upon reasonable request.
